# Microbiology for odour production and abatement

**DOI:** 10.1111/1751-7915.12010

**Published:** 2012-12-18

**Authors:** Lawrence P Wackett

**Affiliations:** Department of Biochemistry, Molecular Biology and Biophysics, BioTechnology Institute, University of MinnesotaSt Paul, MN, 55108, USA

## Sweet smell of microbes

### http://cen.acs.org/articles/90/i29/Sweet-Smell-Microbes.html

This article in an American Chemical Society publication provides a good overview of recent developments in biotechnology for the production of commercially relevant fragrance chemicals.

## Microbial perfume

### http://www.wired.com/wiredscience/2012/07/bacteria-producing-aromas/

This short news article highlights some of the fragrance compounds currently moving toward production and the biotechnology companies that make them using microbial technology.

## Fragrances from microbial oxidations

### http://www.scielo.br/pdf/bjce/v23n3/01.pdf

This review article focuses on specific oxidation reactions catalysed by microorganisms for converting terpene substrates to fragrant, oxygenated products.

## Flavor biotechnology

### http://www.biotecharticles.com/Applications-Article/Flavor-Biotechnology-Part-1-491.html

This web article provides some examples of microbial transformations useful for the production of flavours and fragrances.

## ACS Symposium Series: Fragrances by biotechnology

### http://pubs.acs.org/doi/abs/10.1021/bk-2005-0908.ch004

This page provides a link to a review article that covers fragrance biotechnology in great depth.

## MANE: Flavor and fragrance manufacturer

### http://www.mane.com

This commercial website does not contain much technical information, but it has striking images and illustrates the breadth of the flavour and fragrance market.

## Fine chemicals: Wikipedia

### http://en.wikipedia.org/wiki/Fine_chemical

This general Wikipedia entry on fine chemicals is extensive. It discusses many medium volume chemicals, including flavours and fragrances.

## Industrial microbiology and biotechnology

### http://www.medworm.com/rss/search.php?qu=Journal+of+Industrial+Microbiology+and+Biotechnology&t=Journal+of+Industrial+Microbiology+and+Biotechnology&s=Search&f=source

This page provides a link to a number of interesting microbial transformation reactions, including several used for the purpose of producing flavours and fragrances.

## Allylix: Flavors and fragrances

### http://www.allylix.com/content/flavor-fragrance

Allylix produces sesquiterpene compounds microbially. They describe new processes for producing nootkatone and valencene.

## Flavors and fragrances from fungi

### http://www.fungaldiversity.org/fdp/sfdp/FD13-153-166.pdf

This review article discusses the production of flavours and fragrances using fungi. It has a subtheme on the diversity of reactions catalysed by fungi.

## Novozymes: Microbial odor control

### http://www.novozymes.com/en/solutions/household-care/cleaning-solutions/odor-control/Pages/default.aspx

Novozymes is a major enzyme and biotechnology company that produces a number of biological products for odour control.

## Abora: Microbial odor control

### http://www.aborausa.com/eng/aboramicrobial/odorcontrol.html

This website illustrates the large number of industrial processes that need odour control and provides insight into how that can be accomplished.

## Biofilter, air pollution

### http://en.wikipedia.org/wiki/Biofilter

Biofiltration is a popular method used for treating industrial off gases and odours. This is a general, useful overview of the topic.

